# Reduced aldehyde dehydrogenase expression in preeclamptic decidual mesenchymal stem/stromal cells is restored by aldehyde dehydrogenase agonists

**DOI:** 10.1038/srep42397

**Published:** 2017-02-13

**Authors:** Gina D. Kusuma, Mohamed H. Abumaree, Anthony V. Perkins, Shaun P. Brennecke, Bill Kalionis

**Affiliations:** 1 Department of Obstetrics and Gynaecology, Royal Women’s Hospital, The University of Melbourne, Parkville, Victoria, 3052, Australia; 2 Pregnancy Research Centre, Department of Maternal-Fetal Medicine, Royal Women’s Hospital, Parkville, Victoria, 3052, Australia; 3 Stem Cells and Regenerative Medicine Department, King Abdullah International Medical Research Centre/ College of Science and Health Professions, King Saud Bin Abdulaziz University for Health Sciences, King Abdulaziz Medical City – Ministry of National Guard Health Affairs, P.O. Box 3660, Riyadh 11481, Mail Code 3124, Kingdom of Saudi Arabia; 4 School of Medical Science, Menzies Health Institute Queensland, Griffith University, Gold Coast Campus, Southport, Queensland, 9726, Australia

## Abstract

High resistance to oxidative stress is a common feature of mesenchymal stem/stromal cells (MSC) and is associated with higher cell survival and ability to respond to oxidative damage. Aldehyde dehydrogenase (ALDH) activity is a candidate “universal” marker for stem cells. ALDH expression was significantly lower in decidual MSC (DMSC) isolated from preeclamptic (PE) patients. ALDH gene knockdown by siRNA transfection was performed to create a cell culture model of the reduced ALDH expression detected in PE-DMSC. We showed that ALDH activity in DMSC is associated with resistance to hydrogen peroxide (H_2_O_2_)-induced toxicity. Our data provide evidence that ALDH expression in DMSC is required for cellular resistance to oxidative stress. Furthermore, candidate ALDH activators were screened and two of the compounds were effective in upregulating ALDH expression. This study provides a proof-of-principle that the restoration of ALDH activity in diseased MSC is a rational basis for a therapeutic strategy to improve MSC resistance to cytotoxic damage.

A wide variety of important diseases and disorders are associated with oxidative stress, which include Alzheimer’s disease, angina, atherosclerosis, neurodevelopmental disorders, Parkinson’s disease, thrombosis and hypertension^1^,^2^,^3^. Mesenchymal stem/stromal cells (MSC), introduced topically by intralesional injection, local vascular injection or intravenously, are successful in reducing oxidative stress^4^. Indeed, these data have laid the foundations for many MSC-based human clinical trials^5^,^6^,^7^. However, the use of exogenous MSC is limited by practical problems such as their rarity in tissues and bodily fluids, the need to expand MSC in culture to clinically useful numbers (5 × 10^6 ^per kg) as well as uncertainties in the timing and dosage of MSC, their poor viability in damaged tissues and possible immune reaction^8^. At the root of these problems is our limited understanding of the MSC microenvironment from which MSC are derived, and the mechanism of exogenous MSC repair. Accumulating data show a predominantly paracrine mechanism of action rather than one of MSC engraftment and differentiation^9^. The consistent evidence of a paracrine effect of MSC suggests that exogenous MSC provide soluble factors (e.g. growth factors, cytokines) and biological factors (e.g. exosomes, microvesicles) that allow oxidatively stressed endogenous MSC to avoid apoptosis, resist oxidative stress, proliferate and carry out the necessary repair of diseased tissue^10^. Thus, an alternative MSC therapeutic strategy is to use biological and pharmaceutical agents that improve endogenous MSC survival and other important stem cell functions. Examples of the success of such a strategy was demonstrated when pitavastatin was employed to enhance heme oxygenase-1 expression in MSC, which may protect cells from oxidative stress^11^.

The challenge in devising strategies for reducing oxidative-stresses in endogenous MSC is that obtaining MSC from oxidatively-stressed tissues in sufficient numbers from diseased patients is particularly difficult. This severely limits our understanding of the defective pathways in oxidatively stressed endogenous MSCs and hinders the testing of strategies to improve their function.

Here, we investigate the role of MSC in the hypertensive disorder called preeclampsia (PE). PE is a serious medical disorder of human pregnancy characterised by pregnancy-induced hypertension and proteinuria. If untreated, PE can lead to eclampsia, a convulsive life-threatening disorder. PE results from a series of biological stresses, which lead to abnormal placentation and subsequently affect the mother, fetus and placenta^12^. In PE, the *decidua basalis* is also a major source of reactive oxygen species that cause systemic damage to vascular endothelia^13^. The presence of H_2_O_2_ and superoxide radicals in the placenta and *decidua basalis* leads to lipid peroxidation and results in the production of toxic by-products e.g. lipid hydroperoxides, thiobarbituric acid reactive substances, reactive aldehydes, and malondialdehyde^14^,^15^. Levels of H_2_O_2_ are higher in serum of PE patients and correlate with a state of higher oxidative stress^16^,^17^.

We showed that placental and decidual MSC can be readily isolated following delivery of the baby^18^,^19^. In this study we focus on the *decidua basalis* MSC (DMSC). The *decidua basalis* is essential for the development of the conceptus and for the continuation of normal pregnancy^20^. PE-affected DMSC represent a unique cell model to assess the effects of oxidative stress on MSC, and to test strategies designed to alleviate oxidative stress.

Aldehyde dehydrogenases (ALDH) are a family of enzymes that detoxify aldehydes produced by oxidative stress. Our recent study investigated the immunohistochemical localisation of ALDH in the maternal *decidua basalis* and as expected, ALDH expression was co-localised with the MSC marker, FZD-9, within a vascular niche^19^. Thus, DMSC are proximal to the maternal blood circulation and are directly or indirectly exposed to circulating reactive oxygen species and by-products of oxidative stress. The detoxification capacity of ALDH has the potential to protect stem cells against oxidative damage and is one of the important factors governing their longevity^21^,^22^. Levels of lipid peroxides and malondialdehyde (MDA) are higher in PE-affected *decidua basalis*^23^ and both these products of oxidative stress are implicated in the pathogenesis of PE. To date, the use of antioxidant therapies to target oxidative stress (e.g. vitamin C, vitamin E, coenzyme Q10, carotenoids, and selenium) for women at risk of PE have only showed marginal or little benefits in preventing PE^24^. Thus, other strategies are needed to delay or prevent the onset of PE. The first aim of this study was to determine the level of ALDH enzyme activity between normotensive DMSC and PE-DMSC, and whether ALDH1A1 expression is critical in cellular protection of DMSC against oxidative stress. The other aim of this study was to determine whether ALDH activity could be restored, partially or completely, by treatment with ALDH activators.

## Results

### ALDH expression in DMSC and PE-DMSC

**Figure 1:**
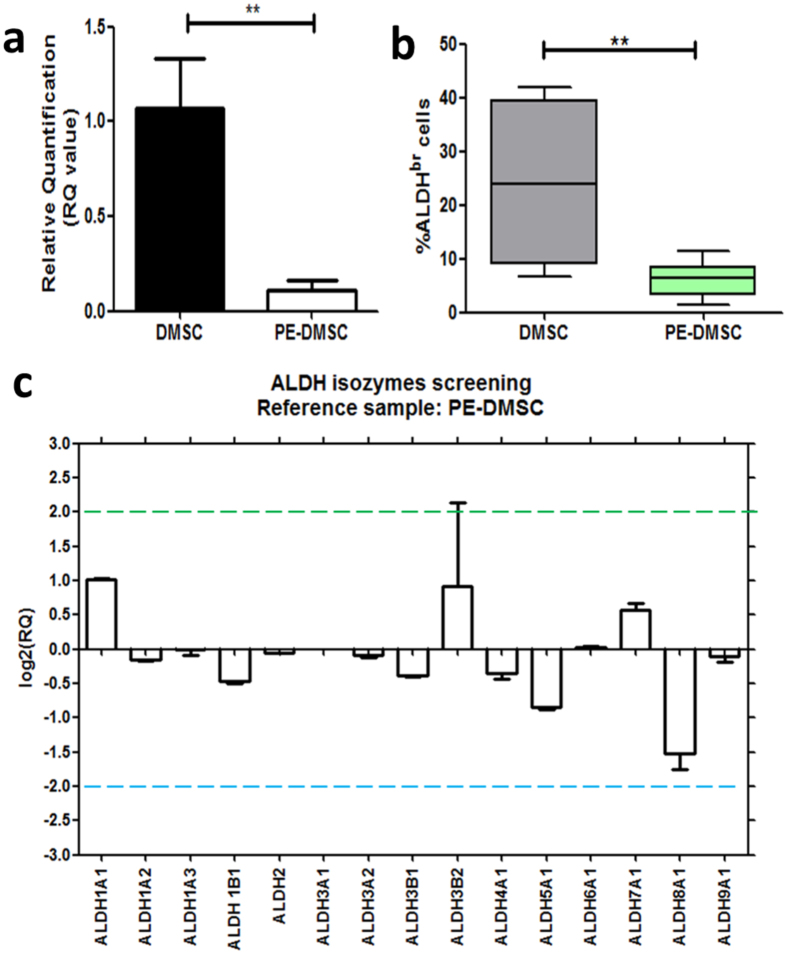
ALDH1A1 mRNA levels and the percentage of ALDH^br^ cells in primary DMSC and PE-DMSC. (**a**) RQ value of ALDH1A1 mRNA levels in primary DMSC and PE-DMSC. Quantitative real-time RT-PCR was performed to determine ALDH1A1 mRNA levels relative to the housekeeping gene 18S rRNA. mRNA levels were then normalised to the reference sample, which was the primary DMSC. Unpaired t-test, n = 6 each group, **p-value = 0.0016. (**b**) The Aldefluor assay results on isolated primary DMSC and PE-DMSC show the percentage of cells with high levels of ALDH enzyme expression (percentage of ALDH^br^ cells). Results are presented as box plots showing the lower quartile, median, upper quartile, and whiskers to depict range. Unpaired t-test, n = 10 each group, **p-value = 0.0013. (**c**) The log_2_(RQ) plot of ALDH isozymes in primary DMSC and PE-DMSC. For each individual gene, the mRNA level was calculated relative to an endogenous control (18S rRNA is the chosen housekeeping gene). Gene expression was then normalised to the calibrator sample, which was the value in the primary PE-DMSC. The Y-axis shows the log_2_ (RQ) values of the particular ALDH isozyme mRNA level. The X-axis shows the ALDH isozymes used in this screening. Genes that are up-regulated have log_2_ (RQ) values > 2 (green line) and down-regulated genes have log_2_ (RQ) values > −2 (blue line). Data are presented as mean ± SEM in duplicate samples.

ALDH1A1 mRNA levels between isolated primary DMSC and PE-DMSC were analysed, with 18S rRNA used as the housekeeping control gene. The fold change or relative quantitation (RQ) value was calculated with reference to the primary DMSC samples. Figure 1a shows significantly lower ALDH1A1 mRNA levels in primary PE-DMSC compared to DMSC (PE-DMSC: 0.11 ± 0.05 vs. DMSC: 1.07 ± 0.27, p-value = 0.0016, n = 6, unpaired t-test).

To verify the differences in ALDH1A1 mRNA levels that were observed from real-time RT-PCR analysis, the Aldefluor assay was employed to quantify ALDH activity in primary DMSC and PE-DMSC. There was a significantly lower proportion of ALDH^br^ cells in primary PE-DMSC compared to DMSC (p-value = 0.0013, n = 10, unpaired t-test) (Fig. 1b). The primary DMSC cell population contained 24.17 ± 4.67% of ALDH^br^ cells whilst PE-DMSC cell population contained 6.10 ± 0.95% of ALDH^br^ cells. Furthermore, we observed qualitatively lower ALDH1 protein expression in PE-DMSC compared with DMSC using immunofluorescence as described in Supplementary Fig. 1.

To determine the relative mRNA levels of ALDH isozymes in primary DMSC and PE-DMSC, Fig. 1c shows log_2_ (RQ) values against the target genes. Primary PE-DMSC were used as the calibrator sample for RQ calculation. ALDH1A1 isozyme showed the greatest fold increase of 1.02 relative to PE-DMSC mRNA level while ALDH8A1 isozyme showed the greatest fold decrease of −1.52 relative to PE-DMSC mRNA level. The C_T_ values are presented in Table 1. C_T_ values < 30 are associated with an abundance of mRNA transcript in the sample, whereas higher C_T_ values (>30) are associated with low levels of mRNA transcript. DMSC and PE-DMSC expressed similar levels of mRNA for the various ALDH isozymes. In primary DMSC, mRNA levels of ALDH1A1 (26.46 ± 0.01), ALDH1B1 (26.46 ± 0.03), and ALDH9A1 (26.51 ± 0.09) isozymes were the highest. The ALDH3A1 isozyme mRNA was only detected in primary DMSC. In primary PE-DMSC, ALDH1B1 (26.25 ± 0.00), ALDH9A1 (26.67 ± 0.06), and ALDH1A1 (27.74 ± 0.04) isozymes were the highest. Overall, taken together with their C_T_ values, mRNA levels of ALDH1A1 and ALDH8A1 isozyme were altered between DMSC and PE-DMSC.

### Generation and validation of the ALDH1A1 gene knockdown model

To create a cell culture model of ALDH1A1 knockdown, the siRNA transfection method was utilised on the transduced DMSC23 cell line. DMSC and PE-DMSC are primary cells and they are inherently difficult to study because of their limited life span *in vitro* and the potential for patient-to-patient variation between preparations of primary cells. The DMSC23 cell line has extended lifespan and maintains the MSC characteristics of the primary cells and therefore was employed for this knockdown model. Supplementary Fig. 2 shows the optimization of ALDH1A1 siRNA transfection performed with various siRNA concentrations (5 nM, 10 nM, and 20 nM). At 5 nM siRNA concentration, none of the siRNAs showed a significant reduction in the ALDH1A1 mRNA levels compared to the NC and mock controls. However, at the 10 nM siRNA concentration, all but si2 showed a significant reduction in the ALDH1A1 mRNA levels. At 20 nM all siRNAs significantly reduced ALDH1A1 mRNA levels compared with the NC and mock controls (NC: 1.01 ± 0.12, mock: 1.23 ± 0.21, vs. si2: 0.24 ± 0.05, si5: 0.41 ± 0.14, si6: 0.26 ± 0.08, and si7 0.16 ± 0.01). The conditions used for consistent and efficient ALDH1A1 gene knockdown were a 72-hour incubation period and a concentration of 20 nM siRNA. Two siRNAs, si6 and si7, were selected for subsequent experiments due to their consistency in reducing ALDH1A1 mRNA levels.

**Figure 2:**
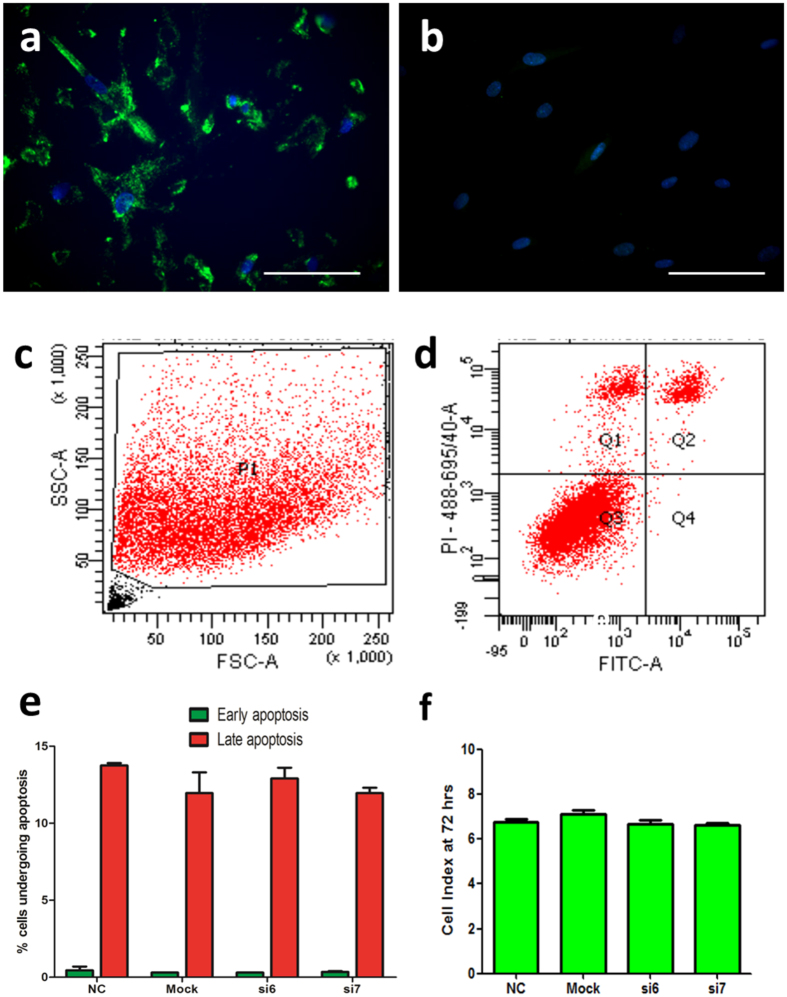
Validation of ALDH1A1-siRNA transfection in DMSC23 cells. (**a**) Validation of siRNA uptake with fluorescent Allstars NC-AF488 siRNA. DMSC23 cells were transfected with a fluorescent (FITC) siRNA, Allstars NC-AF488, to visualise siRNA uptake. DMSC23 cells showing uptake of Allstars NC-AF488 (FITC). (**b**) Control with the omission of Allstars NC-AF488. Reactions were carried out in three independent experiments. Cell nuclei were counter-stained with DAPI (blue). Magnification is 200X and scalebar is 100 μm. (**c**) Example of gating parameters for Annexin V apoptosis assay. FSC versus SSC dot plot showing the gate used for this analysis. Dots in the lower left portion of the plot represent cellular debris. FSC: forward scatter, SSC: side scatter. (**d**). Dual-staining plot for apoptosis detection with Propidium Iodide/PI on the Y-axis and Annexin V/FITC on the X-axis. Events in Q2: PI positive and FITC positive show cells undergoing late apoptosis. Events in Q3: PI negative and FITC negative show viable cells. Events in Q4: PI negative and FITC positive show cells undergoing early apoptosis. (**e**) Quantification of early and late apoptosis using Annexin V staining following ALDH1A1-siRNA transfection of DMSC23 cells. The Y-axis shows the percentage of cells undergoing apoptosis and the X-axis the different sample groups. Data are presented as mean ± SEM from three independent experiments. One-way ANOVA test, p-value = 0.5219 (late apoptosis group), p-value = 0.6396 (early apoptosis group). (**f** ) xCELLigence system proliferation assay following 72-hour ALDH1A1-siRNA transfection of DMSC23 cells. The Y-axis represents the cell index at the 72 hrs timepoint and X-axis represents the different sample groups. Data are presented as mean ± SEM from triplicates of three independent experiments. NC (non-specific siRNA control) and mock (non-siRNA control). One-way ANOVA test, p-value = 0.1496.

The efficiency of the siRNA transfection was evaluated by transfecting the DMSC23 cells with nonsilencing (NC) siRNA which is directly conjugated to AF488 (FITC fluorescence). Figure 2a shows transfection of siRNAs in DMSC23 cells, as evidenced from the FITC fluorescence around the cell nuclei due to the siRNAs being taken up by the cells. More than 90% of the cells were transfected by qualitative assessment of at least three fields of view. The negative control, where Allstars NC-AF488 siRNA was omitted, showed no significant FITC fluorescence surrounding the cell nuclei, which were counterstained with DAPI (Fig. 2b).

Following ALDH1A1-siRNA transfection, Annexin V apoptosis assays were carried out to determine whether ALDH1A1 siRNA knockdown affected cell apoptosis. A representative flow cytometry plot to demonstrate the gating settings for Annexin V assay is shown in Fig. 2c,d. Figure 2e shows the percentage of ALDH1A1 siRNA-transfected DMSC23 cells undergoing both early and late apoptosis and this was not significantly different between the NC, mock, si6- and si7-transfected DMSC23 cells (p > 0.05, n = 3, One-way ANOVA test).

The xCELLigence system was employed to carry out real-time proliferation assays to measure changes in cell proliferation at the end of the 72 hrs siRNA transfection period. Figure 2f presents the CI values of DMSC23 cells treated with siRNAs compared with mock or NC controls. At 72 hrs post-siRNA transfection, the proliferation rates of ALDH1A1 siRNA-transfected DMSC23 cells were not significantly different to either controls (NC: 6.76 ± 0.14, mock: 7.14 ± 0.16, vs. si6: 6.66 ± 0.20, si7: 6.66 ± 0.09, p > 0.05, n = 3, One-way ANOVA test). Thus, the DMSC23 cell proliferation rate was not affected by the ALDH1A1 siRNA transfection.

ALDH isozyme screening was carried out to determine the specificity of the effect of ALDH1A1-siRNA knockdown in DMSC23 cells. The NC samples were used as the calibrator sample to calculate the fold change. To illustrate any positive (i.e. up-regulation) or negative (i.e. down-regulation) effects of ALDH1A1-siRNA on ALDH isozyme mRNA levels, log_2_(RQ) values or fold change were calculated and plotted on the Y-axis against the target ALDH isozyme genes on the X-axis (Supplementary Fig. 3). The screening array identified the ALDH1A1 isozyme with a fold decrease of 2.17 (si6) and 2.26 (si7) in mRNA levels. Other ALDH isozymes did not show changes greater or less than 2-fold. Overall, this finding shows that only the ALDH1A1 isozyme was significantly down-regulated, and therefore the siRNAs were specific for the ALDH1A1 isozyme.

### Molecular characterisation of the ALDH1A1 siRNA transfection

**Figure 3:**
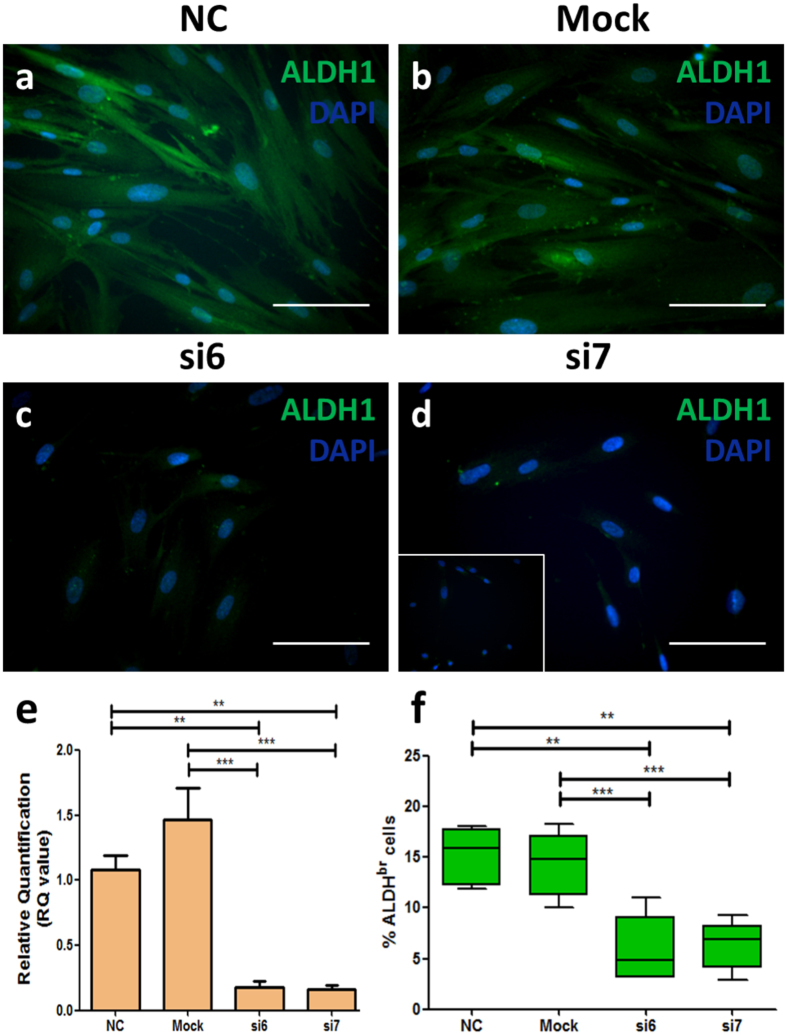
Molecular characterisation of ALDH1A1-siRNA transfected cells. ALDH1 antibody immunocytochemistry on DMSC23 cells following a 72-hour ALDH1A1-siRNA transfection. Cells were incubated with ALDH1-specific antibody (FITC) and then the cell nuclei were counter-stained with DAPI (blue). (**a**) NC-transfected DMSC23 cells, (**b**) Mock control DMSC23 cells, (**c**) si6-transfected DMSC23 cells, and (**d**) si7-transfected DMSC23 cells. Inset shows negative control with the omission of the primary antibody. NC (non-specific siRNA control) and Mock (no added siRNA control). Reactions were carried out in at least three independent experiments. Magnification is 200X and scalebar is 100 μm. (**e**) Quantitative real-time RT-PCR detecting ALDH1A1 mRNA levels relative to the 18S rRNA housekeeping gene. ALDH1A1 transfection was performed with 20 nM siRNA for a 72-hour period. The Y-axis shows the RQ value of ALDH1A1 mRNA levels calculated relative to the NC group as the calibrator. The X-axis shows samples analysed; si6, si7 and mock mRNA levels relative to the NC control. Data are presented as mean ± SEM from duplicates. One-way ANOVA test, n = 5, significant differences are denoted by ***p-value < 0.001, **p-value < 0.01. (**f** ) Aldefluor assay following ALDH1A1-siRNA transfection of DMSC23 cells. The Y-axis shows flow cytometric analysis of the percentage of cells expressing high levels of ALDH (ALDH^br^ cells) while the X-axis shows the different siRNAs used for the ALDH1A1 knockdown, NC, and Mock groups. Results are presented as box plots showing the lower quartile, median, upper quartile, and whiskers to depict range. Data are presented as mean ± SEM. One-way ANOVA test, n = 5, significant differences are denoted by ***p-value < 0.001, **p-value < 0.01.

Changes in ALDH1A1 protein expression was assessed by immunofluorescence detection. The DMSC23 cells were transfected with NC, mock, si6, and si7 and after a 72-hour transfection period, the cells were fixed on chamber slides. ALDH1 antibody showed a strong FITC fluorescence staining in the cell cytoplasm in the NC and mock controls (Fig. 3a,b). In contrast, ALDH1A1-siRNA treated cells showed relatively low FITC fluorescence (Fig. 3c,d). The negative control was the omission of the primary antibody (inset in Fig. 3d). The result suggested that ALDH1 protein expression was lower in ALDH1A1-siRNAs treated DMSC23 cells compared with the NC and mock controls.

The mRNA levels of ALDH1A1 were further assessed using an independent, validated ALDH1A1 Taqman gene probe. Figure 3e shows RQ values derived from real-time RT-PCR analysis with the NC control used as the calibrator sample. Significant down-regulation of ALDH1A1 mRNA levels was confirmed following treatment with ALDH1A1 si6 or si7 compared to the NC and mock controls (NC: 1.08 ± 0.12, Mock: 1.47 ± 0.24 vs. si6: 0.18 ± 0.04, si7: 0.17 ± 0.03, p < 0.001, n = 5, One-way ANOVA test). This was equivalent to 82% and 83% down-regulation by si6 and si7, respectively.

The Aldefluor assay was used to examine the effect of ALDH1A1-siRNA knockdown on the percentage of ALDH^br^ DMSC23 cells, which reflects ALDH enzyme activity. Figure 3f demonstrates that siRNA treated DMSC23 cells had a significantly lower percentage of ALDH^br^ cells compared with the NC and mock controls (NC: 15.18 ± 1.27%, mock: 14.32 ± 1.42% vs. si6: 5.84 ± 1.49%, si7: 6.30 ± 1.07%, p-value < 0.001, n = 5, One-way ANOVA test). Therefore, the ALDH1A1 gene knockdown resulted in a significantly lower ALDH activity in the siRNAs treated DMSC23 cells.

### ALDH1A1 enzyme in cellular protection against oxidative stress

Utilising the xCELLigence-based cytotoxity assay, DMSC23 cells were seeded into each well of a 96-well E-Plate and transfected with different ALDH1A1-siRNAs. During the first 4–6 hrs, cells started to adhere to the bottom of the wells, causing an initial rise in the CI. After 72 hrs transfection, a medium change was performed and H_2_O_2_ was added to induce oxidative stress. From this timepoint, CI was measured hourly for at least 24 hrs. Increasing CI represented actively growing cells whilst decreasing CI represented cell senescence or cell death. The H_2_O_2_ concentrations tested ranged from 100 to 500 μM and a concentration of 250 μM H_2_O_2_ was chosen, because most of the control cells remained viable after this treatment. Normalisation of the cell index (NCI) was performed at the 72-hour timepoint and NCI values for all wells were set to 1.0 at this time point.

**Figure 4:**
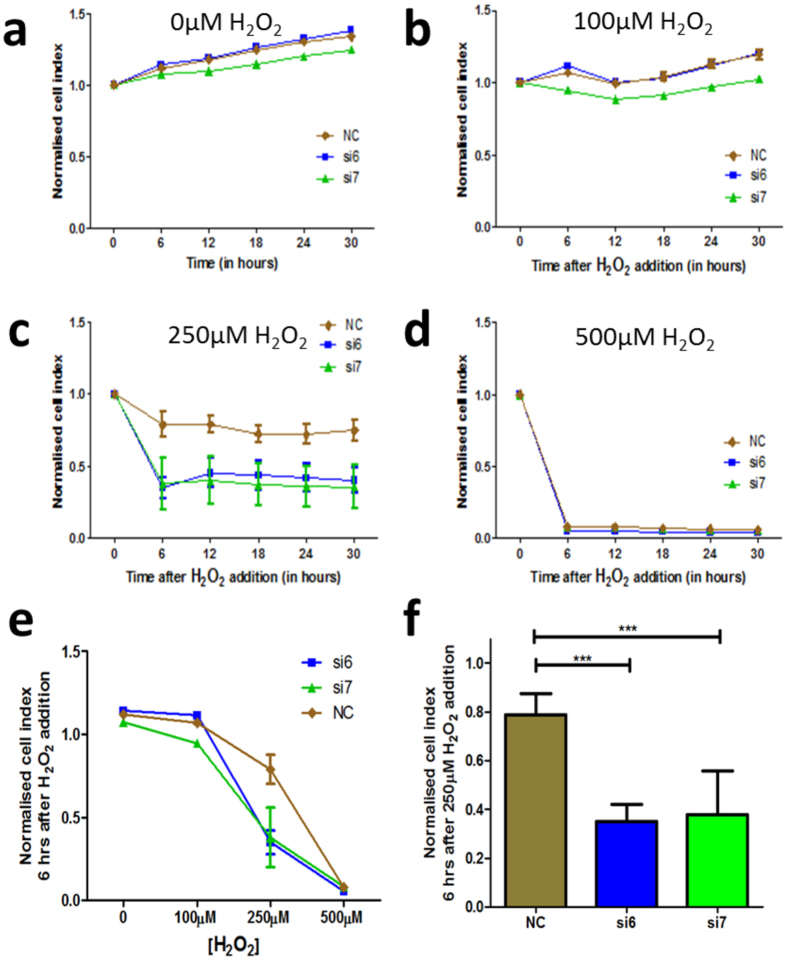
Effect of varying H_2_O_2_ concentration on cell proliferation following ALDH1A1-siRNA transfection of DMSC23 cells. DMSC23 cells transfected with ALDH1A1-siRNAs (si6 and si7) were subjected to (**a**) 0 μM H_2_O_2_ treatment, (**b**) 100 μM H_2_O_2_ treatment, (**c**) 250 μM H_2_O_2_ treatment, and (**d**) 500 μM H_2_O_2_ treatment. The Y-axis shows the normalised cell index (NCI) values and the X-axis shows the time after the initial 72-hour transfection period. (**e**) H_2_O_2_-induced cytotoxicity assay following ALDH1A1-siRNA transfection of DMSC23 cells. The Y-axis shows NCI values and the X-axis the various H_2_O_2_ concentrations added to the cells. (**f** ) The NCI values after 250 μM H_2_O_2_ treatment were plotted. The Y-axis shows NCI values and the X-axis the 6-hour timepoint after the addition of 250 μM H_2_O_2_. Data are presented as mean ± SEM from triplicate samples of three independent experiments. Two-way ANOVA test, n = 3, ***p-value < 0.001.

The effect of H_2_O_2_ treatment after 72 hrs siRNA treatment was determined using the xCELLigence system. At 72 hrs post-siRNA transfection, the control group that did not receive any H_2_O_2_ treatment showed a steady increase in the NCI values (Fig. 4a). Addition of 100 μM H_2_O_2_ treatment resulted in a lower NCI values (see Fig. 4b). Figure 4c shows the NCI values for the experimental group which received 250 μM H_2_O_2_ treatment started to decrease over a period of 24 hrs. On the other hand, the 500 μM H_2_O_2_ treatment showed a cytotoxic effect by killing most of the cells (Fig. 4d).

Figure 4e shows the NCI values at the 6-hour timepoint after various concentrations of H_2_O_2_ treatment between NC and ALDH1A1-siRNA treated DMSC23 cells. The NCI values after 250 μM H_2_O_2_ treatment were plotted in Fig. 4f. The NCI values for NC were significantly higher than those of siRNA treated DMSC23 cells after 250 μM H_2_O_2_ treatment (NC: 0.79 ± 0.09 vs. si6: 0.35 ± 0.07, si7: 0.38 ± 0.18, p-value < 0.001, n = 3, Two-way ANOVA test). Thus, DMSC23 cell proliferation was significantly reduced following 250 μM H_2_O_2_ treatment compared with the NC control.

### Effect of ALDH activator compounds on DMSC23 cells

**Figure 5:**
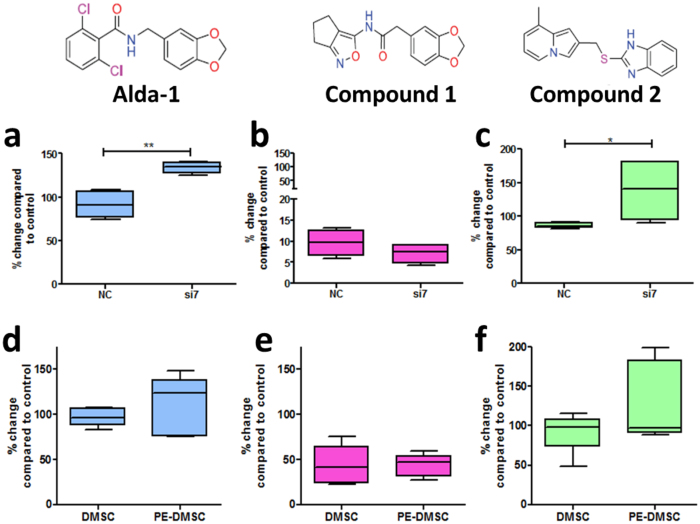
Flow cytometry analysis of ALDH1A1-siRNA transfected DMSC23, primary DMSC and PE-DMSC with the addition of ALDH compounds using Aldefluor assay. Top panel: The Y-axis shows the percentage change of ALDH^br^ cells compared with the control (untreated). The X-axis shows the test groups, NC-transfected and ALDH1A1-siRNA (si7) transfected DMSC23 cells. Bottom panel: The X-axis shows the test groups, primary DMSC and PE-DMSC. Results are presented as box plots showing the lower quartile, median, upper quartile, and whiskers to depict range. (**a** & **d**) The effect of 10 μM Alda-1 on the percentage of ALDH^br^ cells. (**a**) Unpaired t-test, n = 4, **p-value = 0.0023. (**d**) Unpaired t-test, n = 5, p-value = 0.4075. (**b** & **e**) Effect of 1 μM Compound 1 on the percentage of ALDH^br^ cells. (**b**) Unpaired t-test, n = 4, p-value = 0.2342. (**e**) Unpaired t-test, n = 5, p-value = 0.9862. (**c** & **f**) Effect of 10 μM Compound 2 on the percentage of ALDH^br^ cells. (**c**) Unpaired t-test, n = 4, *p-value = 0.0369. (**f**) Unpaired t-test, n = 5, p-value = 0.1852. Data are presented as mean ± SEM from duplicates.

Changes in ALDH activity reflected the effect of ALDH1A1 activator compounds in the siRNA treated DMSC23 cells. Following treatment with 10 μM Alda-1, 1 μM Compound 1, and 10 μM Compound 2, the Aldefluor assay was carried out. Figure 5a shows that the addition of Alda-1 resulted in a significantly higher percentage of ALDH^br^ cells in si7-transfected cells (NC: 91.15 ± 7.86%, si7: 134.10 ± 3.24%, p-value = 0.0023, n = 4, unpaired t-test). Figure 5b shows Compound 1 to have an inhibitory effect as indicated by the lower percentage of ALDH^br^ cells following treatment (NC: 9.65 ± 1.57%, si7: 7.10 ± 1.12%, p-value = 0.2342, n = 4, unpaired t-test). Figure 5c shows addition of Compound 2 resulted in a significantly higher percentage of ALDH^br^ cells in si7-transfected cells (NC: 86.05 ± 2.16%, si7: 138.8 ± 24.31%, p-value = 0.0369, n = 4, unpaired t-test).

### Effect of ALDH activator compounds on primary DMSC

Following treatment with 10 μM Alda-1, 1 μM Compound 1, and 10 μM Compound 2, the Aldefluor assay was carried out on primary DMSC and PE-DMSC. The control for this experiment was the untreated group without any added compound and the data were presented as the percentage change compared to the control. Figure 5d shows Alda-1 treatment did not result in any significant difference in the percentage of ALDH^br^ cells between primary DMSC and PE-DMSC (DMSC: 96.85 ± 4.41%, PE-DMSC: 110.30 ± 14.75%, p-value = 0.4075, n = 5, unpaired t-test). Figure 5e shows Compound 1 did not show any significant difference in the percentage of ALDH^br^ cells between primary DMSC and PE-DMSC (DMSC: 43.50 ± 9.75%, PE-DMSC: 43.30 ± 5.60% p-value = 0.9862, n = 5, unpaired t-test). Figure 5f shows addition of Compound 2 did not result in a significant difference in the percentage of ALDH^br^ cells between primary DMSC and PE-DMSC (DMSC: 92.19 ± 11.37%, PE-DMSC: 128.80 ± 22.54%, p-value = 0.1852, n = 5, unpaired t-test). Thus, in contrast to the previous results with the siRNA treated DMSC23 cells, Alda-1 and Compound 2 did not produce significant differences in the percentage of ALDH^br^ cells in primary PE-DMSC.

**Figure 6:**
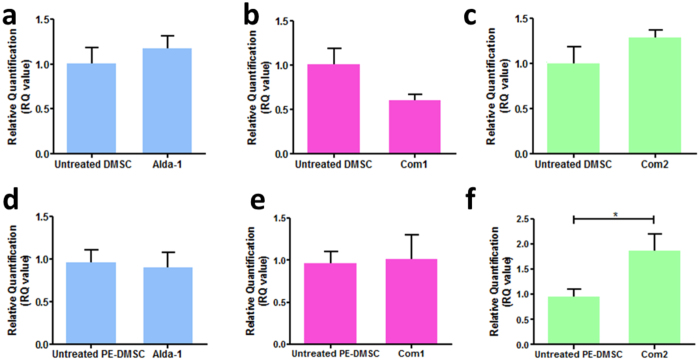
Differences in ALDH1A1 relative mRNA levels following addition of ALDH compounds in primary DMSC and PE-DMSC. (**a**) Untreated (no added compound) primary DMSC were used as the calibrator sample (set to 1.0). The effect of 10 μM Alda-1 on ALDH1A1 relative mRNA level. Unpaired t-test, n = 5, p-value = 0.4683. (**b**) The effect of 1 μM Compound 1 on ALDH1A1 relative mRNA level. Unpaired t-test, n = 5, p-value = 0.0701. (**c**) The effect of 10 μM Compound 2 on ALDH1A1 relative mRNA level. Unpaired t-test, n = 5, p-value = 0.1796. (**d**) Untreated (no added compound) primary PE-DMSC were used as the calibrator sample (set to 1.0). The effect of 10 μM Alda-1 on ALDH1A1 relative mRNA level. Unpaired t-test, n = 5, p-value = 0.4260. (**e**) The effect of 1 μM Compound 1 on ALDH1A1 relative mRNA level. Unpaired t-test, n = 5, p-value = 0.1859. (**f**) Effect of 10 μM Compound 2 on ALDH1A1 relative mRNA level. Unpaired t-test, n = 5, *p-value = 0.0410. The Y-axis shows the RQ value of ALDH1A1 mRNA level relative to the 18S rRNA housekeeping gene. The X-axis shows untreated, 10 μM Alda-1, 1 μM Compound 1, and 10 μM Compound 2 groups. Data are presented as mean ± SEM from duplicates.

Subsequently, ALDH1A1 mRNA levels between the control and treated groups were analysed relative to the housekeeping gene 18S rRNA. The fold change, or RQ value, was calculated with reference to the control (no added compound) samples. Figure 6a–c show that in primary DMSC, treatment with Alda-1, Compound 1, and Compound 2 did not result in significant differences in RQ values (Control: 1.02 ± 0.17, Alda-1: 1.19 ± 0.13, Compound 1: 0.61 ± 0.06, and Compound 2: 1.30 ± 0.08). Figure 6d shows that in primary PE-DMSC, treatment with Alda-1 did not result in significant differences in RQ values (Control: 1.01 ± 0.10 vs. Alda-1: 0.83 ± 0.14, p-value = 0.4260, n = 5, unpaired t-test). Compound 1 treatment also did not show any effect on the ALDH1A1 mRNA level in primary PE-DMSC as indicated in Fig. 6e (Control: 1.01 ± 0.10 vs. Compound 1: 1.28 ± 0.11, p-value = 0.1859, n = 5, unpaired t-test). Figure 6f shows that Compound 2 treatment resulted in a significantly higher ALDH1A1 mRNA level in PE-DMSC (Control: 1.01 ± 0.10 vs. Compound 2: 2.05 ± 0.23, p-value = 0.0410, n = 5, unpaired t-test).

## Discussion

Isolated primary DMSC and PE-DMSC obtained from gestation-matched normotensive and PE patients were analysed for their ALDH expression. Real-time RT-PCR analysis with the ALDH1A1 gene specific probe showed that ALDH1A1 mRNA levels were reduced in primary PE-DMSC. The Aldefluor assay results were consistent in that ALDH enzyme activity was significantly reduced in primary PE-DMSC compared to DMSC. This is the first evidence that PE-DMSC have a reduced capacity to respond to oxidative stress in PE. Defective repair mechanisms in oxidative stress environments are therefore likely to make PE-DMSC more susceptible to apoptosis and reduce their ability to support endothelial cell survival.

In women with PE, levels of oxidative stress are further elevated above those in women with normotensive pregnancies. The *decidua basalis* is a major site of circulating oxidative stress products^13^ and in the case of PE, DMSC in the vascular niche are exposed to these products that cause local and systemic damage to endothelial cells. Chronically lower ALDH enzyme activity, following exposure to circulating oxidative stress products in the *decidua basalis*, could play an important role in the pathophysiology of PE. To model reduced ALDH enzyme activity in PE-DMSC *in vitro*, we measured resistance to oxidative stress after specifically inactivating ALDH1A1 by siRNA inactivation.

The ALDH1A1 enzyme has been studied extensively for its roles in drug resistance and oxidative stress response^21^,^25^,^26^. The present study demonstrated that the ALDH1A1-siRNA transfection method was an effective and specific method to achieve inactivation of ALDH1A1 gene expression in DMSC23 cells. The siRNA effect was seen 72 hrs post-siRNA transfection and the use of ALDH1A1 si6 and si7 at 20 nM resulted in consistent, significantly lower ALDH1A1 mRNA levels. The siRNA treatment did not have any direct effect on DMSC23 cell proliferation and apoptosis as shown by the xCELLigence proliferation assay and Annexin V assay. Therefore, the findings presented here correspond to changes in ALDH1A1 gene expression. We demonstrated that siRNA-treated DMSC23 cells showed significant knockdown of ALDH1A1 mRNA and protein levels, and enzymatic activity. Real-time RT-PCR, immunocytochemistry, and Aldefluor assay were employed to investigate the reduction in ALDH1A1 mRNA, protein, and activity, respectively. The NC and mock controls exhibited similar results, without any significant effect due to ALDH1A1-siRNA knockdown. Subsequently, NC was used as the control further siRNA transfection experiments. The ALDH1A1-siRNA knockdown in DMSC23 cells reproduced the lower ALDH enzyme activity observed in PE-DMSC.

Our ALDH1A1-siRNA knockdown cell model data are consistent with several studies that utilise this gene knockdown method, which show that ALDH1A1 expression is an important factor in the response to oxidative stress. In one study, ALDH1A1-specific siRNA transfection was performed in rat and human lens epithelial cells and the knockdown was associated with higher susceptibility of the cells to oxidative stress associated damage, including apoptosis^25^. In a study of mouse liver, ALDH1A1 enzyme contributed substantially to the oxidation of aldehydes formed by lipid peroxidation^26^. These results suggest that the ALDH1A1 enzyme plays an important physiological role in protecting cells from reactive aldehyde-induced cell death.

We demonstrated for the first time that ALDH1A1-siRNA gene knockdown in DMSC23 cells resulted in a significant reduction in cell viability upon exposure to H_2_O_2_-induced oxidative stress. The ALDH1A1-siRNA treated DMSC23 cells were subjected to cytotoxicity assays to test their resistance to the oxidative stress inducer H_2_O_2_, and the xCELLigence system was used to quantify cell proliferation in real-time. A reduction in NCI values was observed in the ALDH1A1-siRNA treated DMSC23 cells following 250 μM H_2_O_2_ treatment, which corresponds to reduced cell viability. This suggests that ALDH1A1-siRNA DMSC23 cells were oxidatively stressed by the addition of H_2_O_2_; a potent inducer of oxidative stress. This study showed by *in vitro* knockdown experiments in DMSC23 cells, that the ALDH1A1 enzyme plays an important role in the cellular defence against H_2_O_2_–induced toxicity. These findings support those of other studies, which demonstrate that the ALDH1A1 enzyme plays a role in cellular resistance to damage associated with oxidative stress, such as in human neuroblastoma cell lines and human myoblasts^26^,^27^,^28^,^29^,^30^,^31^. Therefore, targeting up-regulation or overexpression of the ALDH1A1 enzyme could be a novel therapeutic strategy to protect against toxic events triggered by oxidative stress.

We showed that ALDH levels were low in PE-DMSC. In an *in vitro* cell model, we showed that lowering ALDH levels reduced the ability of cells to resist oxidative stress. Thus, mechanisms to activate or up-regulate ALDH1A1 gene expression may provide a rationale for therapeutic protection against PE. To test this strategy, in the final aim of the work we used a novel approach to restore resistance to oxidative stress in ALDH1A1-siRNA transfected DMSC23 cells by employing ALDH activators.

Three candidate ALDH1A1 activators (i.e. Alda-1, Compound 1, and Compound 2) were selected based on a published study where ALDH1A1 modulators were identified by a high-throughput screen and their ALDH1A1 activator activity was verified by the DOPAL enzyme assay^32^. These ALDH1A1 activator compounds were used in the ALDH1A1-siRNA transfected DMSC23 to test the ability of these compounds to up-regulate the ALDH1A1 enzyme activity. The Aldefluor assay showed that Alda-1 and Compound 2 significantly increased the percentage of ALDH^br^ cells in the ALDH1A1-siRNA transfected DMSC23 cells. On the other hand, Compound 1 showed no significant activator effect and indeed the percentage of ALDH^br^ cells in both the NC and ALDH1A1-siRNA transfected DMSC23 cells was reduced after treatment. Compound 1 is an ALDH1A1 activator in the DOPAL inactivation assay, which measures the ability of the compound to inactivate ALDH1A1 *in vitro*. This study showed Compound 1 did not produce the same activator effect on ALDH1A1 enzyme expression in the DMSC23 cell culture model. Our findings reflect the complexity of a cell culture model that resulted in a different outcome from the DOPAL assay. This study provides the first evidence that Alda-1 and Compound 2 can up-regulate ALDH1A1 enzyme expression in our cell culture model.

ALDH activator compounds were added to primary DMSC and PE-DMSC, to establish whether the ALDH1A1 activators had a similar effect in primary DMSC and PE-DMSC, to that seen in the ALDH1A1-siRNA transfected DMSC23 cell culture model. The Aldefluor assay data (Fig. 5d–f) showed Alda-1 and Compound 2 did not result in a higher percentage of ALDH^br^ cells in PE-DMSC following addition of these compounds. Compound 1 had an inhibitory effect with a significant reduction in the percentage of ALDH^br^ cells in both primary DMSC and PE-DMSC following treatment. However, real-time RT-PCR data analysis provided evidence that Compound 2 significantly increased ALDH1A1 relative mRNA levels in primary PE-DMSC. Alda-1 and Compound 1 treatment did not show any significant changes in ALDH1A1 mRNA levels in either primary DMSC or PE-DMSC. In summary, these findings provide evidence that ALDH activator compounds can increase ALDH enzyme activity and/or mRNA levels in both the DMSC23 cells and primary PE-DMSC.

Alda-1 is the most extensively studied of the three compounds, since it is also an activator of the ALDH2 enzyme^33^. Alda-1 treatment reduces infarct size in rodent models of ischemic heart damage, most likely through its inhibitory effect on the formation of cytotoxic aldehydes^34^. In another animal study of myocardial infarct injury, the heart pathology was inhibited when the drug nitroglycerin was administered with Alda-1^35^. In another study, ALDH2 activation by Alda-1 ameliorated ischemia or reperfusion injury *in vivo*, suggesting that ALDH2 activator compounds may be useful to patients with acute myocardial infarction, cardiac bypass surgery or heart transplantation^36^. Therefore, the application of ALDH1A1 activator compounds represents a feasible approach to the treatment of other oxidative stress-related pathologies such as PE.

The results of testing ALDH activators in this study were consistent with results of recent studies in Parkinson’s disease. The ALDH1A1 enzyme was reported to be down-regulated in brain tissues affected by Parkinson’s disease and researchers investigated the role of ALDH1A1 in mediating HNE toxicity^37^. Another study showed that addition of 6-methyl-2-(phenylazo)-3-pyridinol, an ALDH1A1 activator, protected neuronal cell lines against HNE-mediated toxicity, demonstrating the protective effect of ALDH1A1 against oxidative stress. These observations are consistent with the notion that higher ALDH1A1 enzyme activity results in higher HNE clearance^27^. Further work needs to be carried out to establish whether 6-methyl-2-(phenylazo)-3-pyridinol could also up-regulate ALDH1A1 enzyme activity in PE-DMSC.

In summary, this study explores a novel strategy whereby upregulating ALDH1A1 expression with ALDH1A1 activators may restore the ability of DMSC to respond to oxidative stress. Such activator compounds may ultimately have therapeutic potential in the treatment of PE. Restoring resistance to oxidative stress in PE-DMSC could allow these stem cells to resume important functions, including repair of metabolic pathways, vascular support and differentiation. ROS and by-products of oxidative stress manifest via multiple pathways and consequently, there are multiple means through which protection can be conferred. There are several therapeutic established interventions for reducing oxidative stress. For example, the use of aldehyde scavengers aminoguanidine and tenilsetam to reduce the reactive aldehydes and protect neuroblastoma cells against methylglyoxal toxicity^38^. The results of this study should encourage the identification of more potent ALDH1A1 activators and subsequent testing in cell culture models and preclinical animal models of PE.

## Methods

### Collection of normotensive and preeclamptic placenta tissues

Placentae from pregnancies complicated by PE (n = 10) and gestation-matched normotensive pregnancies (n = 10) were collected. Informed and written consent was obtained from all patients and the study was approved by the Royal Women’s Hospital Human Research Ethics Committee. All clinical procedures were carried out in accordance with the National Health and Medical Research Council guidelines. The placentae were collected after Caesarean section or vaginal delivery. PE was diagnosed when new onset hypertension (blood pressure greater than 140/90 mm Hg occurred after 20 weeks’ gestation and was accompanied by new onset proteinuria of greater than 0.3 g/24 hour^39^. All PE samples collected for these experiments were gestation-matched to healthy, normotensive patients and the detailed patient characteristics are shown in Table 2.

### Cell culture

DMSC were isolated from the *decidua basalis* that remained attached to the placenta using previously published methods^18^,^20^. DMSC were cultured in α-MEM medium (Sigma-Aldrich) with 10% FBS, 100 U/ml penicillin, 100 mg/ml streptomycin and 2 mM L-Glutamine (Sigma Aldrich).

DMSC23 cell lines were created by human telomerase reverse transcriptase (hTERT) transduction of the primary DMSC^40^. DMSC23 cells were maintained in culture using Mesencult Proliferation Kit (Stem Cell Technologies) which consists of Mesencult basal medium, 10% Mesencult supplement, 100 U/ml penicillin, and 100 mg/ml streptomycin. All cell cultures were maintained at 37 °C with 5% CO_2_ in a humidified incubator. DMSC and DMSC23 cells were characterised by flow cytometry for expression of CD90, CD146, CD166, CD44, CD73, CD105 and were negative for CD45, CD19, and HLA-DR. The cells displayed multi-lineage differentiation potential along the osteogenic, adipogenic, and chondrogenic lineages as shown previously^18^,^19^,^40^. Primary cell and cell lines were passaged after reaching 80% confluency. At each passage, cells were harvested using TrypLE Express solution (Life Technologies) and cultured on uncoated tissue culture flasks. All cultured primary cells were used for experiments up to P5 and DMSC23 cell lines up to P30.

### Real-time RT-PCR

To quantify ALDH1A1 mRNA levels, cell pellets were collected and stored for RNA extraction, followed by cDNA synthesis and real-time RT-PCR. RNA was isolated using the Purelink RNA mini kit (Life Technologies) according to the manufacturer’s instructions. The total RNA concentration and integrity was determined using the Nanodrop 2000c (Thermo Scientific). Following RNA extraction, a two-step reverse transcriptase (RT) PCR method was performed to synthesise cDNA from 2 μg RNA with 500 ng random primers and 0.5 mM of dNTP mix followed by incubation for 5 mins at 65 °C. Subsequently, 1 μl DTT, 40U RNasin Plus RNase inhibitor, 200U of Superscript III reverse transcriptase and 4 μl of 5X First Strand buffer was added. The samples were placed in the GeneAmp PCR system 9700 using the following cycling conditions: 25 °C for 5 mins, 50 °C for 60 mins and 70 °C for 15 mins. PCR reaction was performed with ALDH1A1 Taqman gene (FAM probe, Hs00946916_m1) primers in a duplex reaction with 18S rRNA housekeeping gene (VIC probe, Hs99999901_s1). The reaction was carried out in the ABI 7500 (Applied Biosystems) using the following cycling conditions: 10 mins at 95 °C, 95 °C for 15 secs and 60 °C for 1 min, repeated for 40 cycles. Changes in gene expression were calculated by the comparative C_T_ method and fold differences were calculated using 2^−ΔΔCt^ method^41^.

### Real-time RT-PCR ALDH isozymes screening

Pooled cDNA samples (n = 5) were used in a custom made Taqman array (Applied Biosystems) containing primers of known ALDH isozymes, and 18S rRNA as an endogenous control gene. Details of individual Taqman probes are provided in Supplementary Table 1. PCR reaction was carried out as described in previous section.

### Aldefluor assay

The flow cytometry-based Aldefluor assay (Stem Cell Technologies) was employed to quantify cells with high level expression of ALDH (i.e. ALDH^br^ cells) in PE-DMSC and DMSC. Cells were suspended in Aldefluor assay buffer and incubated for 30 mins according to manufacturer’s instructions. In each experiment, cells incubated with the ALDH inhibitor, diethylaminobenzaldehyde (DEAB), served as a negative control. The amount of intracellular fluorescence was measured by flow cytometry using BD LSRII and analysed using FACS Diva software (BD Biosciences).

### ALDH1A1 siRNA transfection

ALDH1A1 gene was inactivated by siRNA transfection in the DMSC23 cell line. 24 hrs prior to transfection, 1 × 10^5^ DMSC23 cells were seeded in each well of a 6-well plate in Mesencult medium. Cells were transfected with 20 nM ALDH1A1-specific siRNA in HiPerfect transfection reagent for 72 hrs. Details of the siRNAs are presented in Supplementary Table 2. As controls, Allstars NC siRNA (Qiagen) and mock control (HiPerfect reagent only) were used in each experiment. Initially, transfection efficiency was determined by seeding cells overnight and then transfecting them with fluorescently labelled Allstars NC-AF488 (Qiagen). Uptake of siRNA by DMSC23 cells was detected by FITC fluorescence microscopy and was present around cell nuclei.

### Determination of siRNA effects on cell apoptosis and proliferation

To determine whether siRNA treatment had any effects on cell apoptosis and proliferation, the following assays were used. Early and late cell apoptosis in ALDH1A1-siRNA transfected DMSC23 cells was measured by Annexin V staining (BD Biosciences). Cells were selected for flow cytometric analysis based on their forward (FSC) versus side scatter (SSC) profile. The Annexin V assay kit (Annexin V/Propidium Iodide staining) allows the detection of early and late stages of apoptotic cells. Viable cells with intact membranes will exclude propidium iodide (PI) but apoptotic/dead cells will take up PI. Early apoptotic cells (Annexin V positive, PI negative) are undergoing apoptosis but they maintain membrane integrity. Late apoptosis, or end stage apoptotic cells (Annexin V positive, PI positive) are considered to have undergone apoptotic death. Measurement of the effect of ALDH1A1-siRNA transfection on the DMSC23 cell proliferation was carried out using the xCELLigence system. Cells were plated at a density of 1 × 10^4^ cells into an E-Plate 96 in the xCELLigence MP system (ACEA Biosciences). The impedance in each well was monitored every 15 mins during initial attachment for the first 4 hrs and thereafter was measured every hour, for 72 hrs. The cell index (CI) values were obtained from the RTCA 2.0 software (ACEA Biosciences).

### Molecular characterisation of the ALDH1A1 siRNA transfection

Verification of the ALDH1A1 gene knockdown was conducted at the mRNA, protein, and functional levels. mRNA levels were determined by real-time RT-PCR with ALDH1A1 Taqman gene probe as described earlier. Immunocytochemistry with the ALDH1-specific antibody was performed to examine changes in ALDH1 protein expression after siRNA transfection in cultured cells. Cells were fixed with 70% ethanol and blocked with 5% skim milk powder for 1 hr at RT and then washed with PBS. ALDH expression was determined by staining with mouse anti-human ALDH1 (BD Biosciences) followed by donkey anti-mouse Alexa Fluor 488 (Life Technologies) secondary antibody. The negative control was omission of the primary antibodies. Nuclear counterstaining was carried out by the addition of Vectashield mounting medium with DAPI (Vector Laboratories). Fluorescence microscopy was visualised on an Olympus IX81 microscope and the resulting images were compiled by Cell R software (Olympus). At the functional level, the Aldefluor assay was used to measure changes in ALDH activity following ALDH1A1-siRNA transfection.

### ALDH1A1 enzyme in cellular protection against oxidative stress

The cytotoxicity assay was carried out using the xCELLigence system to determine if ALDH1A1-transfected DMSC23 cells were more susceptible to oxidative stress damage. Using the E-Plate 96, 1 × 10^4^ cells were seeded in each well containing 200 μl Mesencult medium. Cells were transfected with 20 nM siRNAs together with the HiPerfect transfection reagent. At the end of the 72-hour transfection period, H_2_O_2_ was added to the wells, initially at different concentrations ranging from 100 μM–500 μM to establish the cytotoxicity effect. The impedance in each well was monitored using the xCELLigence system and the CI was measured every hour for at least 48 hrs. Assays were performed in triplicate and culture medium-only controls were used for the background reading. Normalised cell index (NCI) values were calculated at the 72-hour timepoint, which was the endpoint of the ALDH1A1-siRNA transfection period. For each well, the NCI was calculated as the CI at a given timepoint divided by the CI at the normalisation timepoint, as described in eq. (1). Therefore, the NCI for all wells must equal one at the normalisation timepoint.


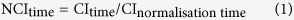


### Compound selection

The first high-throughput compound screening for ALDH1A1 activators was carried out by the DOPAL (3,4-dihydroxyphenylacetaldehyde) inactivation assay^27^,^32^. The screen produced three candidate ALDH compounds; Alda-1, Compound 1, and Compound 2, which were selected for this study based on their activity, potency, and commercial availability. Alda-1 was purchased from Sigma-Aldrich; Compound 1 and Compound 2 were purchased from Molport (Riga, Latvia). Compound stock solutions were prepared as follows: Alda-1 stock solution as 10 mM dissolved in DMSO, Compound 1 and Compound 2 stock solutions as 50 mM dissolved in DMSO. The IUPAC name of each compound is described below:

**Alda**-**1**: N-(1,3-Benzodioxol-5-ylmethyl)-2,6-dichlorobenzamide.

**Compound 1**: 2-(2H-1,3-benzodioxol-5-yl)-N-(4H,5H,6H-cyclopenta[c][1,2]oxazol-3-yl)acetamide.

**Compound 2**: 2-[((8-methylimidazo[1,2-a]pyridin-2-yl)methyl)sulfanyl]-1H-1,3-benzodiazole.

### Aldefluor assay with compounds treatment

The ALDH1A1-siRNA transfection was performed in a 6-well plate and after a 72-hour incubation period, the media was changed and the following treatments, 10 μM Alda-1, 1 μM Compound 1, and 10 μM Compound 2 were added to the cells. The compound concentrations were chosen following preliminary experiments to generate a concentration curve (data not shown). Following a 24-hour incubation period with these compounds, cells were trypsinised and analysed by the Aldefluor assay to determine the level of ALDH activity. A control group was included, which contained siRNA treated DMSC23 cells without any added compound. The data was presented as the percentage change compared with the no added compound control. The data were calculated as described on eqs (2–7):


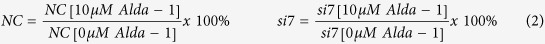



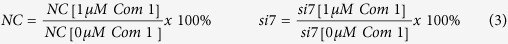



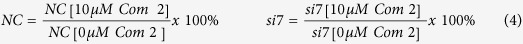



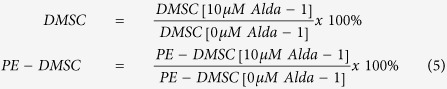



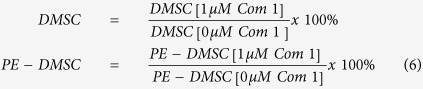



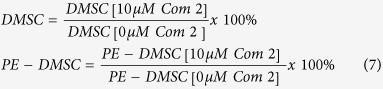


### Statistical analysis

All data were described as mean ± SEM. All experiments were carried out in triplicate, unless otherwise stated. An unpaired student t-test was used to assess the difference in clinical features, the difference in percentage of ALDH^br^ cells, and real-time RT-PCR results between different groups. The one-way ANOVA test was used to determine statistical significance between si6-, si7-, NC-transfected, and the mock control groups. A two-way ANOVA test was used in the cytotoxicity test as there were two factors being tested, i.e. the different transfection groups and H_2_O_2_ concentrations. A p-value of <0.05 was considered significant. Statistical calculations were performed using the GraphPad Prism software.

## Additional Information

**How to cite this article:** Kusuma, G. D. *et al*. Reduced aldehyde dehydrogenase expression in preeclamptic decidual mesenchymal stem/stromal cells is restored by aldehyde dehydrogenase agonists. *Sci. Rep.* **7**, 42397; doi: 10.1038/srep42397 (2017).

**Publisher's note:** Springer Nature remains neutral with regard to jurisdictional claims in published maps and institutional affiliations.

## Supplementary Material

Supplementary Information

## Figures and Tables

**Table 1:** C_T_ values from primary DMSC and PE-DMSC.

Gene	DMSC	PE-DMSC	log_2_(RQ)
ALDH1A1	26.46 ± 0.01#	27.74 ± 0.04	1.02 ± 0.01
ALDH1A2	28.76 ± 0.01	28.69 ± 0.03	−0.15 ± 0.01
ALDH1A3	32.97 ± 0.07	33.22 ± 0.18	−0.01 ± 0.07
ALDH1B1	26.46 ± 0.03	26.25 ± 0.00	−0.47 ± 0.03
ALDH2	28.74 ± 0.01	28.96 ± 0.01	−0.05 ± 0.01
ALDH3A1	36.90 ± 0.12	40.00 ± 0.00	0.00 ± 0.00
ALDH3A2	27.39 ± 0.03	27.55 ± 0.05	−0.10 ± 0.03
ALDH3B1	28.18 ± 0.02	28.06 ± 0.07	−0.38 ± 0.02
ALDH3B2	35.67 ± 1.22	36.84 ± 0.32	0.92 ± 1.22
ALDH4A1	28.86 ± 0.08	28.77 ± 0.07	−0.35 ± 0.08
ALDH5A1	30.52 ± 0.02	29.93 ± 0.02	−0.85 ± 0.03
ALDH6A1	28.30 ± 0.02	28.58 ± 0.06	0.02 ± 0.02
ALDH7A1	28.05 ± 0.10	28.80 ± 0.16	0.58 ± 0.10
ALDH8A1	35.11 ± 0.23	33.85 ± 0.04	−1.52 ± 0.23
ALDH9A1	26.51 ± 0.09	26.67 ± 0.06	−0.10 ± 0.09

^#^C_T_ values reflect the fractional cycle number at which the fluorescence intensity exceeds the threshold intensity. Data are presented as mean ± SEM.

**Table 2:** Normotensive and PE patient characteristics.

Patient Characteristics*		Normotensive (n = 10)	PE (n = 10)	p-value
Maternal age (years)		30.40 ± 1.19	31.00 ± 1.93	0.79
Maternal BMI		25.47 ± 1.19	31.09 ± 2.46	0.06
Gestational age (weeks)		39.00 ± 0.33	38.40 ± 0.40	0.26
Mode of delivery	Vaginal	1	3	
	Caesarean	9	7	
Infant sex	Male	7	7	
	Female	3	3	
Birth weight (g)		3627 ± 185.2	3236 ± 132.2	0.10
Placental weight (g)		693 ± 32.32	679.6 ± 41.06	0.80
Mean systolic bp (mmHg)		109.2 ± 1.78	156.7 ± 1.93	<0.0001
Mean diastolic bp (mmHg)		66.68 ± 1.70	98.35 ± 1.49	<0.0001
Proteinuria (g/24 hrs)		0	0.59 ± 0.14	<0.001

^*^All parameters of the gestation-matched normotensive and PE pregnancies were described as mean ± SEM.

## References

[b1] Kurozumi K. . Mesenchymal stem cells that produce neurotrophic factors reduce ischemic damage in the rat middle cerebral artery occlusion model. Mol Ther 11, 96–104 (2005). 15585410 10.1016/j.ymthe.2004.09.020

[b2] Sayre L. M. . 4-Hydroxynonenal-derived advanced lipid peroxidation end products are increased in Alzheimer’s disease. J. Neurochem. 68, 2092–7 (1997). 9109537 10.1046/j.1471-4159.1997.68052092.x

[b3] Finkel T. & Holbrook N. J. Oxidants, oxidative stress and the biology of ageing. Nature 408, 239–247 (2000). 11089981 10.1038/35041687

[b4] Valle-Prieto A. & Conget P. a. Human mesenchymal stem cells efficiently manage oxidative stress. Stem Cells Dev. 19, 1885–1893 (2010). 20380515 10.1089/scd.2010.0093

[b5] Phinney D. G. & Prockop D. J. Concise Review: Mesenchymal Stem/Multipotent Stromal Cells: The State of Transdifferentiation and Modes of Tissue Repair—Current Views. Stem Cells 25, 2896–2902 (2007). 17901396 10.1634/stemcells.2007-0637

[b6] English K., French A. & Wood K. J. Mesenchymal stromal cells: facilitators of successful transplantation? Cell Stem Cell 7, 431–442 (2010). 20887949 10.1016/j.stem.2010.09.009

[b7] Baksh D., Song L. & Tuan R. S. Adult mesenchymal stem cells: characterization, differentiation, and application in cell and gene therapy. J Cell Mol Med 8, 301–316 (2004). 15491506 10.1111/j.1582-4934.2004.tb00320.x PMC6740223

[b8] Timmins N. E. . Closed system isolation and scalable expansion of human placental mesenchymal stem cells. Biotechnol. Bioeng. 109, 1817–1826 (2012). 22249999 10.1002/bit.24425

[b9] Liu S. H. . Paracrine factors from human placental multipotent mesenchymal stromal cells protect endothelium from oxidative injury via STAT3 and manganese superoxide dismutase activation. Biol Reprod 82, 905–913 (2010). 20107204 10.1095/biolreprod.109.081828

[b10] Dey R. . Human mesenchymal stem cells increase anti-oxidant defences in cells derived from patients with Friedreich’s ataxia. Cerebellum 11, 861–71 (2012). 22826109 10.1007/s12311-012-0406-2

[b11] Kawashiri M.-A. . Impact of Enhanced Production of Endogenous Heme Oxygenase-1 by Pitavastatin on Survival and Functional Activities of Bone Marrow-derived Mesenchymal Stem Cells. J. Cardiovasc. Pharmacol. 65, 601–6 (2015). 25714596 10.1097/FJC.0000000000000231 PMC4461382

[b12] Redman C. W. G. Stress responses and pre-eclampsia. Pregnancy Hypertens. An Int. J. Women’s Cardiovasc. Heal. 3, 57 (2013). 10.1016/j.preghy.2013.04.003 26105836

[b13] Staff A. C., Dechend R. & Pijnenborg R. Learning from the placenta: acute atherosis and vascular remodeling in preeclampsia-novel aspects for atherosclerosis and future cardiovascular health. Hypertension 56, 1026–1034 (2010). 20956732 10.1161/HYPERTENSIONAHA.110.157743

[b14] Raijmakers M. T., Peters W. H., Steegers E. A. & Poston L. Amino thiols, detoxification and oxidative stress in pre-eclampsia and other disorders of pregnancy. Curr Pharm Des 11, 711–734 (2005). 15777228 10.2174/1381612053381837

[b15] Hung T.-H. & Burton G. J. Hypoxia and reoxygenation: a possible mechanism for placental oxidative stress in preeclampsia. Taiwan. J. Obstet. Gynecol. 45, 189–200 (2006). 17175463 10.1016/S1028-4559(09)60224-2

[b16] Aris A., Benali S., Ouellet A., Moutquin J. M. & Leblanc S. Potential Biomarkers of Preeclampsia: Inverse Correlation between Hydrogen Peroxide and Nitric Oxide Early in Maternal Circulation and at Term in Placenta of Women with Preeclampsia. Placenta 30, 342–347 (2009). 19223072 10.1016/j.placenta.2009.01.003

[b17] Kharfi A., Giguère Y., De Grandpré P., Moutquin J. M. & Forest J. C. Human chorionic gonadotropin (hCG) may be a marker of systemic oxidative stress in normotensive and preeclamptic term pregnancies. Clin. Biochem. 38, 717–721 (2005). 15904911 10.1016/j.clinbiochem.2005.04.011

[b18] Kusuma G. D. . Ectopic Bone Formation by Mesenchymal Stem Cells Derived from Human Term Placenta and the Decidua. PLoS One 10, e0141246 (2015). 26484666 10.1371/journal.pone.0141246 PMC4618923

[b19] Kusuma G. D. . Mesenchymal Stem/Stromal Cells Derived From a Reproductive Tissue Niche Under Oxidative Stress Have High Aldehyde Dehydrogenase Activity. Stem Cell Rev. Reports 12, 285–97 (2016). 10.1007/s12015-016-9649-5 26880140

[b20] Kusuma G. D. . Mesenchymal stem cells reside in a vascular niche in the decidua basalis and are absent in remodelled spiral arterioles. Placenta 36, 312–321 (2015). 25575436 10.1016/j.placenta.2014.12.014

[b21] Singh S. . Aldehyde dehydrogenases in cellular responses to oxidative/electrophilicstress. Free Radic. Biol. Med. 56, 89–101 (2013). 23195683 10.1016/j.freeradbiomed.2012.11.010 PMC3631350

[b22] Moreb J. S. Aldehyde dehydrogenase as a marker for stem cells. Curr Stem Cell Res Ther 3, 237–246 (2008). 19075754 10.2174/157488808786734006

[b23] Siddiqui I. A., Jaleel A., Tamimi W. & Al Kadri H. M. Role of oxidative stress in the pathogenesis of preeclampsia. Arch Gynecol Obs. 282, 469–474 (2010). 10.1007/s00404-010-1538-6 20549510

[b24] Roes E. M. . Deficient detoxifying capacity in the pathophysiology of preeclampsia. Med Hypotheses 55, 415–418 (2000). 11058421 10.1054/mehy.2000.1079

[b25] Choudhary S. . Role of aldehyde dehydrogenase isozymes in the defense of rat lens and human lens epithelial cells against oxidative stress. Invest Ophthalmol Vis Sci 46, 259–267 (2005). 15623782 10.1167/iovs.04-0120

[b26] Weiner H. . Murine hepatic aldehyde dehydrogenase 1a1 is a major contributor to oxidation of aldehydes formed by lipid peroxidation. Chem. Biol. Interact. 191, 278–287 (2011). 21256123 10.1016/j.cbi.2011.01.013 PMC4409133

[b27] Kong D. & Kotraiah V. Modulation of aldehyde dehydrogenase activity affects (+/−)-4-hydroxy-2E-nonenal (HNE) toxicity and HNE-protein adduct levels in PC12 cells. J. Mol. Neurosci. 47, 595–603 (2012). 22170038 10.1007/s12031-011-9688-y

[b28] Moreb J. S., Mohuczy D., Ostmark B. & Zucali J. R. RNAi-mediated knockdown of aldehyde dehydrogenase class-1A1 and class-3A1 is specific and reveals that each contributes equally to the resistance against 4-hydroperoxycyclophosphamide. Cancer Chemother Pharmacol 59, 127–136 (2007). 16614850 10.1007/s00280-006-0233-6

[b29] Moreb J. S. . Retinoic acid down-regulates aldehyde dehydrogenase and increases cytotoxicity of 4-hydroperoxycyclophosphamide and acetaldehyde. J Pharmacol Exp Ther 312, 339–345 (2005). 15470086 10.1124/jpet.104.072496

[b30] Jean E. . Aldehyde dehydrogenase activity promotes survival of human muscle precursor cells. J Cell Mol Med 15, 119–133 (2011). 19840193 10.1111/j.1582-4934.2009.00942.x PMC3822499

[b31] Zhang M. . Overexpression of aldehyde dehydrogenase 1A1 reduces oxidation-induced toxicity in SH-SY5Y neuroblastoma cells. J Neurosci Res 88, 686–694 (2010). 19774675 10.1002/jnr.22230

[b32] Kotraiah V., Pallares D., Toema D., Kong D. & Beausoleil E. Identification of aldehyde dehydrogenase 1A1 modulators using virtual screening. J Enzym. Inhib Med Chem 28, 489–494 (2012). 10.3109/14756366.2011.653353 22380773

[b33] Perez-Miller S. . Alda-1 is an agonist and chemical chaperone for the common human aldehyde dehydrogenase 2 variant. Nat Struct Mol Biol 17, 159–164 (2010). 20062057 10.1038/nsmb.1737 PMC2857674

[b34] Chen C.-H. . Activation of aldehyde dehydrogenase-2 reduces ischemic damage to the heart. Science 321, 1493–1495 (2008). 18787169 10.1126/science.1158554 PMC2741612

[b35] Sun L., Ferreira J. C. & Mochly-Rosen D. ALDH2 activator inhibits increased myocardial infarction injury by nitroglycerin tolerance. Sci Transl Med 3, 107ra111 (2011). 10.1126/scitranslmed.3002067 PMC3547591 22049071

[b36] Budas G. R., Disatnik M. H. & Mochly-Rosen D. Aldehyde dehydrogenase 2 in cardiac protection: a new therapeutic target? Trends Cardiovasc Med 19, 158–164 (2009). 20005475 10.1016/j.tcm.2009.09.003 PMC2856486

[b37] Wey M. C. . Neurodegeneration and motor dysfunction in mice lacking cytosolic and mitochondrial aldehyde dehydrogenases: implications for Parkinson’s disease. PLoS One 7, e31522 (2012). 22384032 10.1371/journal.pone.0031522 PMC3284575

[b38] Ellis E. M. Reactive carbonyls and oxidative stress: potential for therapeutic intervention. Pharmacol Ther 115, 13–24 (2007). 17570531 10.1016/j.pharmthera.2007.03.015

[b39] National High Blood Pressure Education Program Working Group on High Blood Pressure in, P. Report of the National High Blood Pressure Education Program Working Group on High Blood Pressure in Pregnancy. Am. J. Obstet. Gynecol. 183, s1–s22 (2000). 10920346

[b40] Qin S. Q. . Establishment and characterization of fetal and maternal mesenchymal stem/stromal cell lines from the human term placenta. Placenta 39, 134–146 (2016). 26992686 10.1016/j.placenta.2016.01.018

[b41] Livak K. J. & Schmittgen T. D. Analysis of relative gene expression data using real-time quantitative PCR and the 2(-Delta Delta C(T)) Method. Methods 25, 402–408 (2001). 11846609 10.1006/meth.2001.1262

