# Supplementary material for: Reduced aldehyde dehydrogenase expression in preeclamptic decidual mesenchymal stem/stromal cells is restored by aldehyde dehydrogenase agonists
Source: Sci Rep. 2017 Feb 13;7:42397. doi: 10.1038/srep42397 (PMC5304324; doi:10.1038/srep42397)

Reduced aldehyde dehydrogenase expression in preeclamptic decidual mesenchymal stem/stromal cells is restored by aldehyde dehydrogenase agonists

Gina D. Kusuma[a,b], Mohamed H. Abumaree[c], Anthony V. Perkins[d], Shaun P. Brennecke[a,b], Bill Kalionis[a,b*]

Affiliations:

[a] Department of Obstetrics and Gynaecology, Royal Women's Hospital, The University of Melbourne, Parkville, Victoria, Australia, 3052

[b] Pregnancy Research Centre, Department of Maternal-Fetal Medicine, Royal Women's Hospital, Parkville, Victoria, Australia, 3052

[c] Stem Cells and Regenerative Medicine Department, King Abdullah International Medical Research Centre/ College of Science and Health Professions, King Saud Bin Abdulaziz University for Health Sciences, King Abdulaziz Medical City – Ministry of National Guard Health Affairs, P.O. Box 3660, Riyadh 11481, Mail Code 3124, Kingdom of Saudi Arabia

[d] School of Medical Science, Menzies Health Institute Queensland, Griffith University, Gold Coast Campus, Southport, Queensland, Australia 9726

Corresponding author:

Dr. Bill Kalionis

Pregnancy Research Centre

Department of Maternal-Fetal Medicine,

Royal Women’s Hospital

Parkville, Victoria, Australia, 3052

Bill.kalionis@thewomens.org.au

Phone: +613 8345 3748

Supplementary Materials and Methods

ALDH1 immunocytochemistry on cultured cells

DMSC and PE-DMSC were fixed with 70% ethanol into an 8-well chamber slide (Ibidi) for immunocytochemistry. To prevent non-specific staining, sections were blocked with 5% skim milk powder for 1 hr at RT and then washed with 1X PBS. ALDH expression was determined by staining with mouse anti-human ALDH1 (BD Biosciences) followed by Alexa Fluor 488 (Life Technologies). Nuclear counterstaining was carried out by the addition of Vectashield mounting medium with DAPI. Fluorescence staining was visualized on an Olympus IX81 microscope with the appropriate fluorescence filters. The resulting multi-colour images were compiled by Cell R software (Olympus).

Optimization and selection of ALDH1A1-specific siRNA

The protocol for ALDH1A1 siRNA transfection is outlined in Materials and Methods. Initially, optimization experiments were conducted to compare the four independent ALDH1A1-siRNAs (siRNA details are listed in Supplemental Table 1) and their efficiency at reducing ALDH1A1 mRNA expression. Then, siRNA concentrations of 5nM, 10nM, and 20nM were tested according to manufacturer's instructions and compared by real-time RT-PCR analysis, to select the siRNA(s) that gave at least 80% knockdown. All experiments were conducted on DMSC23 cells grown in the Mesencult basal medium with supplements and were carried out in triplicate.

Real-time RT-PCR analysis of ALDH1A1-siRNA effects on ALDH isozymes

ALDH isozyme screening was conducted on ALDH1A1-siRNA transfected DMSC23 cells with a custom-made Taqman array plate (see Supplemental Table 2). There were three experimental groups: NC-, si6- and si7-transfected DMSC23 cells with n=4 in each group. cDNA samples from each group were pooled and reactions carried out in duplicate. The calibrator for the RQ calculation was the NC-transfected DMSC23 cells.

Supplementary Tables

Table S1. Individual primers used for real-time RT-PCR ALDH isozyme screening.

Gene symbol	Gene description	Taqman gene expression assay ID
ALDH1A1	aldehyde dehydrogenase 1 family, member A1	Hs00946916_m1
ALDH1A2	aldehyde dehydrogenase 1 family, member A2	Hs00180254_m1
ALDH1A3	aldehyde dehydrogenase 1 family, member A3	Hs00167476_m1
ALDH1B1	aldehyde dehydrogenase 1 family, member B1	Hs00265114_s1
ALDH2	aldehyde dehydrogenase 2 family (mitochondrial)	Hs01007998_m1
ALDH3A1	aldehyde dehydrogenase 3 family, member A1	Hs00964880_m1
ALDH3A2	aldehyde dehydrogenase 3 family, member A2	Hs00166066_m1
ALDH3B1	aldehyde dehydrogenase 3 family, member B1	Hs00997594_m1
ALDH3B2	aldehyde dehydrogenase 3 family, member B2	Hs02511514_s1
ALDH4A1	aldehyde dehydrogenase 4 family, member A1	Hs00186689_m1
ALDH5A1	aldehyde dehydrogenase 5 family, member A1	Hs00542449_m1
ALDH6A1	aldehyde dehydrogenase 6 family, member A1	Hs00194421_m1
ALDH7A1	aldehyde dehydrogenase 7 family, member A1	Hs00609622_m1
ALDH8A1	aldehyde dehydrogenase 8 family, member A1	Hs00988965_m1
ALDH9A1	aldehyde dehydrogenase 9 family, member A1	Hs00997881_m1
18S	Eukaryotic 18S rRNA	Hs99999901_s1

Table S2. The four independent ALDH1A1-siRNAs (designated as si2, si5, si6, and si7) obtained from Qiagen (GeneBank accession numbers NM_000689).

siRNA	Accession numbers	Target sequence
si2	Hs_ALDH1A1_2	CCACGTGGCATCTTTAATAAA
si5	Hs_ALDH1A1_5	TTGAGCGGGCTAAGAAGTATA
si6	Hs_ALDH1A1_6	TTGGCTGATTTAATCGAAAGA
si7	Hs_ALDH1A1_7	CAGGGCCGTACAATACCAATT

Supplementary Figure Legends

Supplementary Figure 1. ALDH1 protein expression in DMSC and PE-DMSC.

Immunofluorescence of DMSC (a-c) and PE-DMSC (d-f). Cells were labelled with ALDH1 antibody (detected with FITC fluorescence) and nuclei were counterstained with DAPI (blue fluorescence). Magnification is 200X and scalebar is 100 µm.

Supplementary Figure 2. Optimization of siRNA concentration for ALDH1A1-siRNA transfection.

Changes in ALDH1A1 relative mRNA levels following ALDH1A1 gene knockdown performed with different siRNA concentrations on DMSC23 cells. (a) 5nM siRNA concentration, (b) 10nM siRNA concentration and (c) 20nM siRNA concentration. The Y-axis shows the relative quantification (RQ) value of ALDH1A1 mRNA relative to the 18S rRNA housekeeping gene. The X-axis shows the four independent siRNAs chosen for the ALDH1A1 knockdown, the NC (non-specific siRNA control) and mock (non-siRNA control). Data are presented as mean ± SEM from duplicate samples in three independent experiments.

Supplementary Figure 3. The $\log_2$(RQ) plot of ALDH isozymes in ALDH1A1-siRNA transfected DMSC23 cells.

Real-time RT-PCR array was performed for ALDH isozyme screening. For each individual ALDH isozyme, mRNA levels were calculated relative to the 18S rRNA housekeeping gene.

Fold change values were subsequently calculated for si6-transfected DMSC23 cells (a) and si7-transfected DMSC23 cells (b). Values were relative to the NC control and were plotted on the Y-axis. Data are presented as mean ± SEM from duplicate samples.

Supplementary Figures

Supplementary Figure 1.

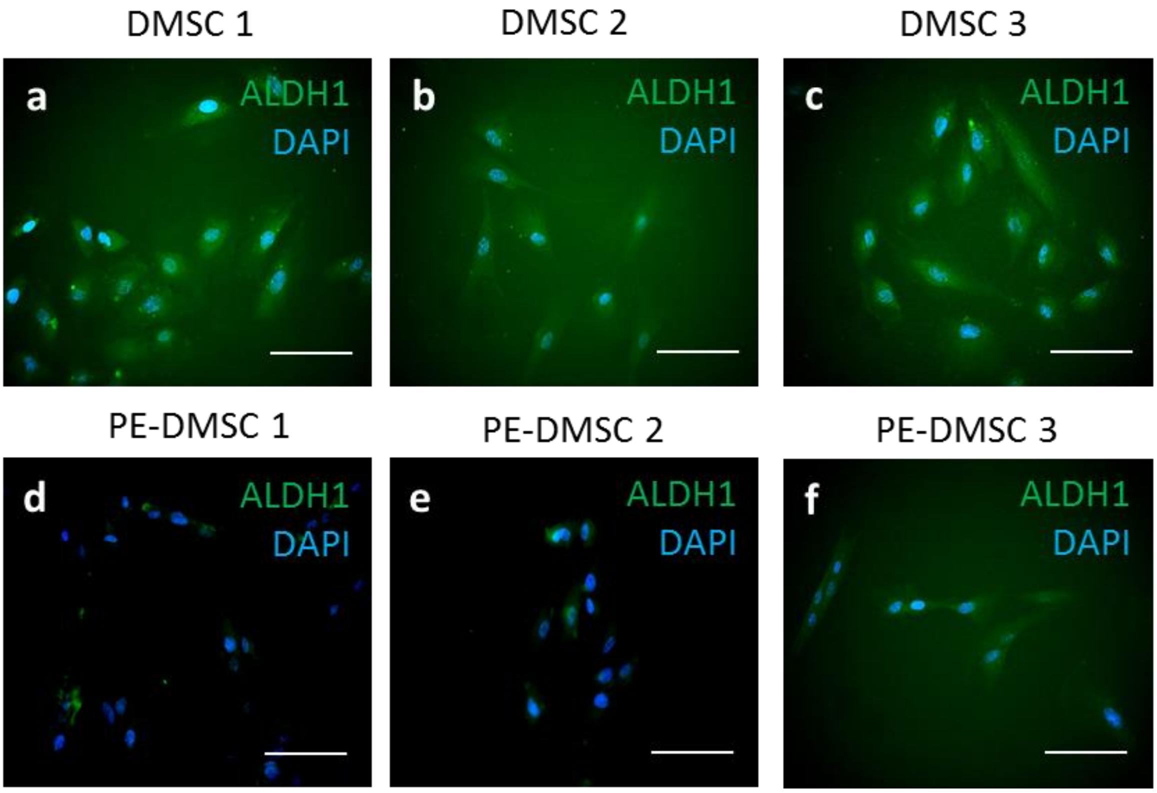

Supplementary Figure 2.

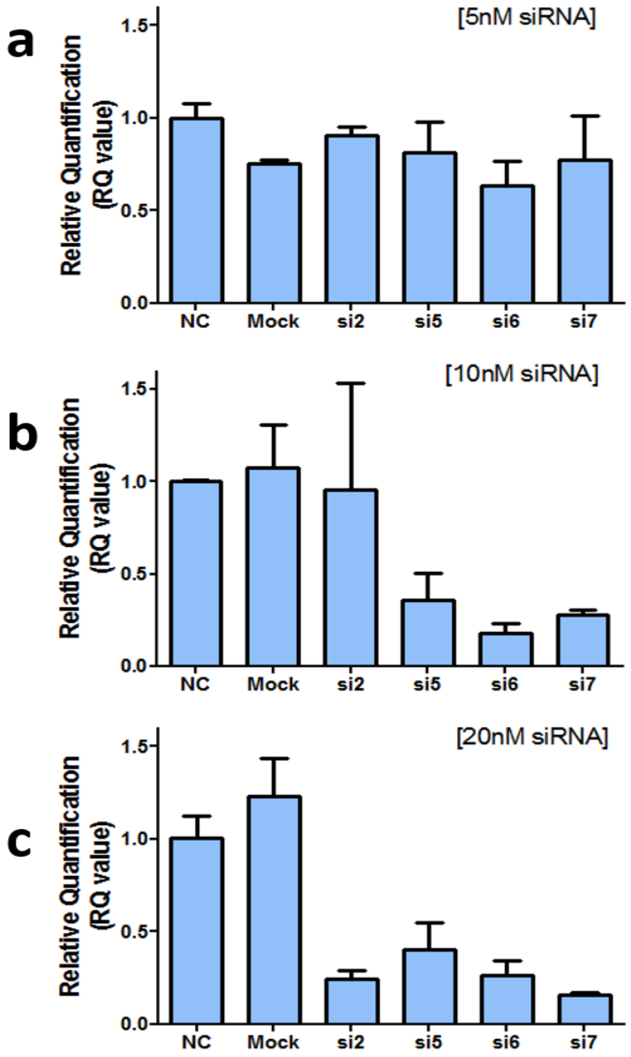

Supplementary Figure 3.

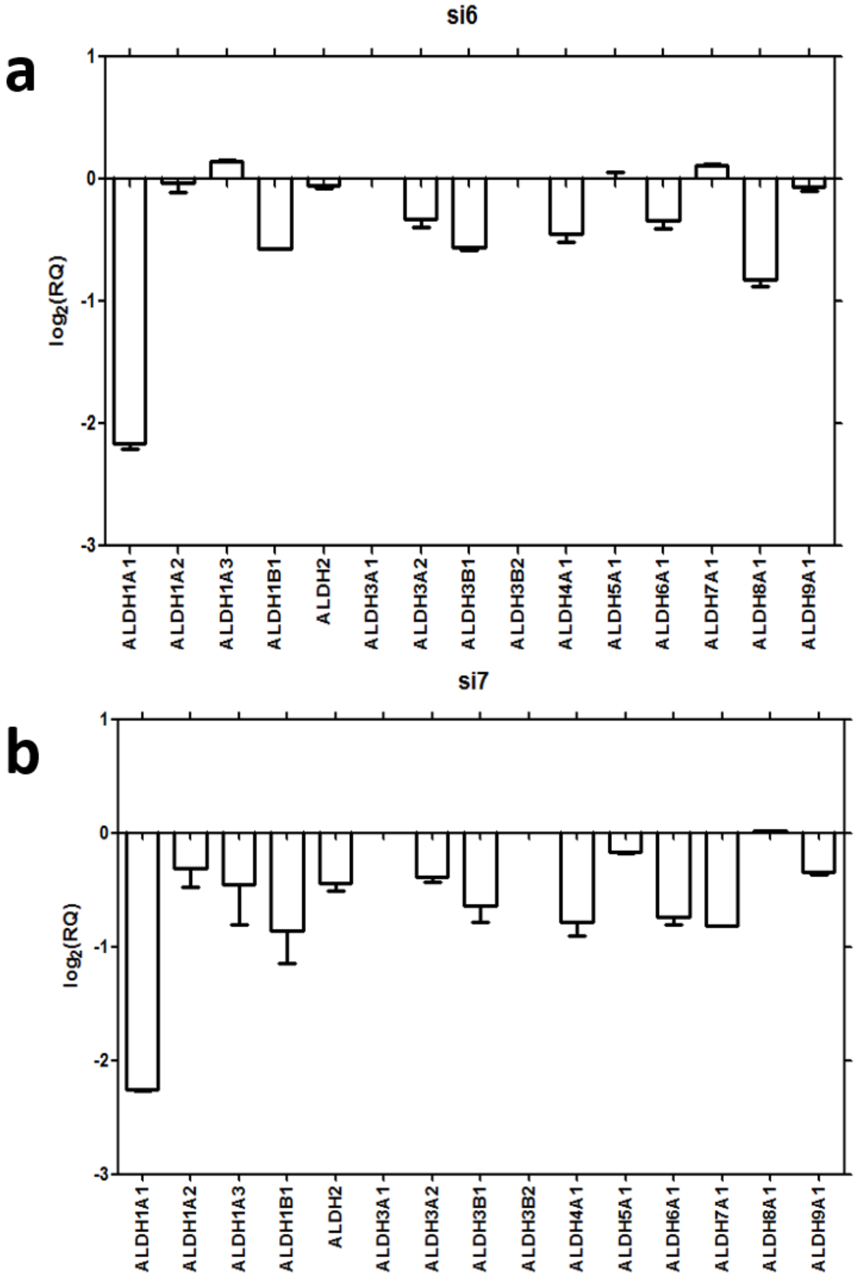

Supplement: Supplementary Information [file srep42397-s1.pdf]
